# RUNX inhibitor suppresses graft‐versus‐host disease through targeting *RUNX‐NFATC2* axis

**DOI:** 10.1002/jha2.230

**Published:** 2021-05-19

**Authors:** Hirohito Kubota, Tatsuya Masuda, Mina Noura, Kana Furuichi, Hidemasa Matsuo, Masahiro Hirata, Tatsuki R. Kataoka, Hidefumi Hiramatsu, Takahiro Yasumi, Tatsutoshi Nakahata, Yoichi Imai, Junko Takita, Souichi Adachi, Hiroshi Sugiyama, Yasuhiko Kamikubo

**Affiliations:** ^1^ Department of Pediatrics Graduate School of Medicine Kyoto University Sakyo‐ku Kyoto Japan; ^2^ Department of Human Health Sciences Graduate School of Medicine Kyoto, University, Sakyo‐ku Kyoto Japan; ^3^ Department of Diagnostic Pathology Kyoto University Hospital Sakyo‐ku Kyoto Japan; ^4^ Drug Discovery Technology Development Office Center for iPS cell research and application (CiRA) Kyoto University Sakyo‐ku Kyoto Japan; ^5^ Department of Hematology/Oncology IMSUT Hospital The Institute of Medical Science The University of Tokyo Tokyo Japan; ^6^ Department of Chemistry Graduate School of Science Kyoto University Sakyo‐ku Kyoto Japan

**Keywords:** graft‐versus‐host disease, NFATC2, polyamide, RUNX

## Abstract

Patients with refractory graft‐versus‐host disease (GVHD) have a dismal prognosis. Therefore, novel therapeutic targets are still needed to be identified. Runt‐related transcriptional factor (RUNX) family transcription factors are essential transcription factors that mediate the essential roles in effector T cells. However, whether RUNX targeting can suppress, and GVHD is yet unknown. Here, we showed that RUNX family members have a redundant role in directly transactivating *NFATC2* expression in T cells. We also found that our novel RUNX inhibitor, Chb‐M’, which is the inhibitor that switches off the entire RUNX family by alkylating agent–conjugated pyrrole‐imidazole (PI) polyamides, inhibited T‐cell receptor mediated T cell proliferation and allogenic T cell response. These were designed to specifically bind to consensus RUNX‐binding sequences (TGTGGT). Chb‐M’ also suppressed the expression of *NFATC2* and pro‐inflammatory cytokine genes in vitro. Using xenogeneic GVHD model, mice injected by Chb‐M’ showed almost no sign of GVHD. Especially, the CD4 T cell was decreased and GVHD‐associated cytokines including tissue necrosis factor‐α and granulocyte‐macrophage colony‐stimulating factor were reduced in the peripheral blood of Chb‐M’ injected mice. Taken together, our data demonstrates that RUNX family transcriptionally upregulates *NFATC2* in T cells, and *RUNX‐NFATC2* axis can be a novel therapeutic target against GVHD.

## 1 INTRODUCTION

The most common life‐threatening complication of hematopoietic stem cell transplantation (HSCT) is graft‐versus‐host disease (GVHD). Approximately 20%–60% of HSCT recipients develop GVHD despite advances in immunosuppressive therapy. In addition, the prognosis for patients with steroid‐refractory acute GVHD (SR‐aGVHD) is dismal, with a survival rate of only 5%‐30% [1]. Ruxolitinib has been shown to be beneficial for SR‐aGVHD, but the overall response rate is still 40% [2], necessitating other novel strategies to control SR‐aGVHD [3, 4].

Nuclear factor of activated T cells (NFAT) is a family of transcription factors identified in activated T cells and promotes the expression of multiple cytokines and other regulatory molecules [5]. Among NFAT family proteins, NFATC2 is a positive regulator of T helper type 1 (Th1) inflammation [6] and has been recognized as a therapeutic target for aGVHD [7]. Calcineurin inhibitors, such as tacrolimus and cyclosporin, inhibit T cell activation, expansion, and effector function by binding to calcineurin and subsequently inhibiting NFAT‐dependent transcription [8]. More specific NFAT‐targeting inhibitors have been developed and validated, but have yet to be analyzed in suitable animal models for potential disease improvement [6, 9].

In addition, proinflammatory cytokines exert direct effects on aGVHD target tissues [10]. Although Th2 and Th17 subsets are also involved in pathogenesis; the predominance of Th1 subsets is well established in mediating aGVHD pathogenesis [11]. Interleukin‐2 (IL‐2), interferon‐γ (IFN‐γ) and tissue necrosis factor‐α (TNF‐α) are the critical cytokines produced during Th1 differentiation and are important in aGVHD [12]. Granulocyte‐macrophage colony‐stimulating factor (GM‐CSF also known as *CSF2*) plays an emerging role across a range of inflammatory diseases [13, 14]. Recent studies have suggested that donor‐derived GM‐CSF is a crucial driver of inflammation and a key factor of determining the severity of the GVHD [15]^–^[17]. Cytokine management for the prophylaxis and treatment of GVHD appears promising, but further efforts are needed to optimize it [18].

The RUNX family of transcription factors (RUNX1, RUNX2, RUNX3) are essential for diverse functions in mammalian cells and regulate the transcription of target genes by recognizing a core consensus DNA‐binding sequence classically referred to as 5′‐TGTGGT‐3′ [19]. RUNX1 and RUNX3 are widely expressed in T cells, in which they play essential roles in T cell development and the acquisition of T cell effector functions [20], and regulate directly *IL2* [21], *IFNG* [22] and *CSF2* [23]. RUNX and NFAT proteins as transcription partners bind in a sequence‐specific manner to the promoter and other regulatory elements of target genes and they work together to regulate target genes [24, 25]. In addition, it has been reported that RUNX2 activates *NFATC2* gene expression in human mesenchymal cells [26], but whether *NFATC2* is regulated by RUNX family in T cells is largely unknown.

We have reported that chlorambucil (Chb)‐conjugated pyrrole‐imidazole (PI) polyamides, Chb‐M’, as a novel RUNX inhibitor which could specifically recognize and bind to RUNX binding sites inhibit RUNX‐mediated gene expression [27, 28], but therapeutic strategies targeting the regulation of gene expression in T cells by RUNX inhibitors have not yet been reported. Based on the assumption of the presence of functional redundancy among the RUNX family members [29], we hypothesize targeting whole‐RUNX family by Chb‐M’ can down‐regulate NFATC2 and pro‐inflammatory cytokine genes of T cells, and reduce GVHD severity.

In this report, we show evidence that inhibition of whole RUNX family inhibits *NFATC2* expression, T cell proliferation and cytokine expression. Furthermore, we examine that RUNX‐inhibitor, Chb‐M’, ameliorates xenogeneic GVHD. Our findings suggest that RUNX inhibition therapy by Chb‐M’ can be a novel therapeutic strategy toward GVHD.

## 2 METHODS

### 2.1 Cell cultures and reagents

The human Jurkat cell line (clone JE6.1) was obtained from The European Collection of. Authenticated Cell Cultures (ECACC, UK, Cat No: 88042803). Embryonic kidney derived HEK293T cell was obtained from Japanese Collection of Research Bioresources (JCRB, Japan). Human peripheral blood was obtained from normal adults following proper consent. Human peripheral blood mononuclear cells (PBMCs) were separated using Lymphoprep (Axis‐Shields, Oslo, Norway) density centrifugation and washed with phosphate‐buffered saline. Jurkat cells and human PBMCs were cultured in RPMI 1640 medium supplemented with 10% fetal calf serum (FCS) and 1% penicillin/streptomycin (P/S), and HEK293T cells were maintained in Dulbecco's modified Eagle's medium (DMEM) supplemented with 10% FCS and 1% P/S at 37°C in a humidified incubator with 5% CO2. For cytokine analysis, cells were stimulated with 50 ng/ml PMA (Wako, Osaka, Japan) and 1μM ionomycin (Sigma‐Aldrich, St. Louis, MO, USA) for 4 h in human PBMCs and 12 h in Jurkat cells, or human PBMCs were also stimulated with anti‐CD3 mAb (OKT3)‐coated plates for 24 hours. For proliferation assay, human PBMC were stimulated by incubation with OKT3‐coated plates in presence of 100 U/ml human recombinant interleukin‐2 (rIL‐2) for 4 days.

### 2.2 Xenogeneic GVHD model

Mice used in this study were 8‐ to 12‐week‐old‐male NOD/Shi‐scid, IL‐2RγKO (NOG) mice. All animal experiments were carried out under protocols approved by Institute of Laboratory Animals of Graduate School of Medicine, Kyoto University. Mice received an intravenous injection of fresh, human PBMCs (5 ×10 [6] cells) from a uniform healthy donor source once on day 0 of the transplant. Mice were treated with Chb‐M’ (320 μg/kg body weight, twice per week intravenous injections) or with the equivalent amount of either chlorambucil (Chb), Chb‐S or DMSO twice a week from day 0. Mice were monitored for GVHD clinical scores, weight and premoribund status. GVHD severity was assessed by a scoring system that incorporates five clinical parameters: weight loss, posture (hunching), activity, fur texture and skin integrity as previously reported [30]. Each parameter received a score of 0 (minimum) to 2 (maximum). Mice that had lost > 20% of their original body weight were euthanized. The final scores of the animals that reached the ethical limit score were kept in the dataset for the remaining time points. Peripheral blood were collected and analyzed by flow cytometry at week 4 after transplantation. To remove red blood cells, blood was lysed in RBC lysing buffer (BD Biosciences) to obtain a cell suspension of mononuclear cells. As indicated, mice were euthanized at 7 weeks after transplantation to study the GVHD pathologic findings of recipient lungs and livers. GVHD pathology scores for recipient liver and lung were assigned in a standard way [31, 32].

### 2.3 Study approval

All animal studies were properly conducted in accordance with the Regulation on Animal Experimentation at Kyoto University, based on International Guiding Principles for Biomedical Research Involving Animals. All procedures employed in this study were approved by Kyoto University Animal Experimentation Committee (Permit Number: Med Kyo 14332).

Additional details for methods sections are provided in the Supplementary Information.

## 3 RESULTS

### 3.1 Redundant roles of RUNX family members in *NFATC2* expression of T cells

**FIGURE 1:**
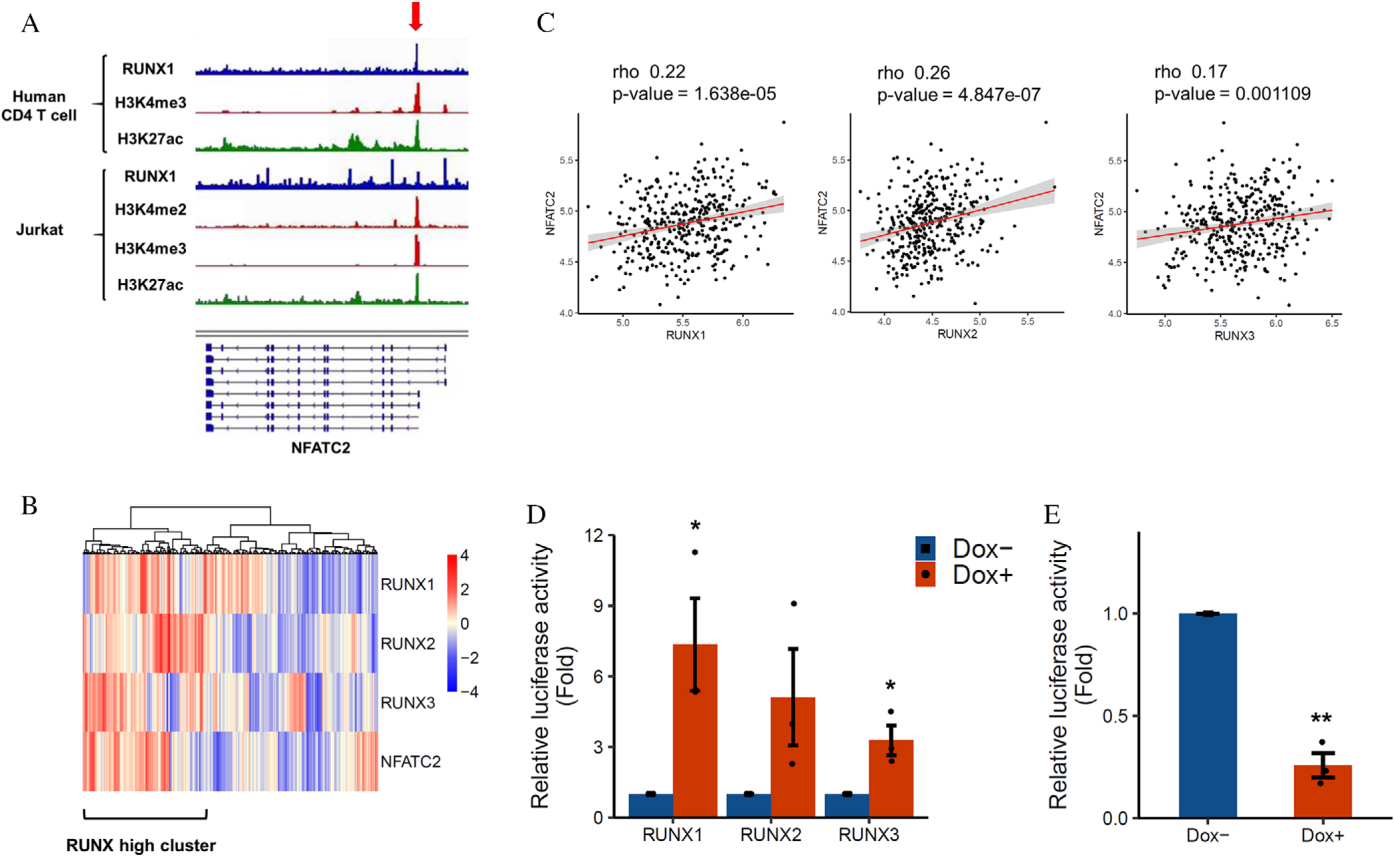
RUNX family‐dependent expression of *NFATC2* in human T cells. (A) IGV snapshots showing the alignment data (BigWig or Wig format) around the *NFATC2* gene loci for RUNX1, H3K4me2, H3K4me3, and H3K27ac ChIP‐seq experiments in primary human CD4 Tcell and Jurkat T cell. Red arrow indicates a region of active chromatin mark in supporting gene activation. (B) Hierarchical clustering with heat map of human CD4 T cell samples (*n* = 376) based on *RUN1, RUNX2, RUNX3* and *NFATC2* gene expression data. (C) Scatter plots showing the correlation of gene expression between *RUNX* family genes and *NFATC2* in human CD4 T cell samples. *p*‐values were calculated by Spearman's correlation. (D and E) Luciferase reporter assay with *NFATC2* promoter. (D) HEK293T cells were stably transduced with the lentivirus expressing *RUNX1, RUNX2* or *RUNX3*, and (E) Jurkat cells were stably transduced with the lentivirus expressing sh*PanRUNX*, together with the reporter vector expressing luciferase gene under *NFATC2* promoter. Cells were incubated with 3 μM doxycycline (Dox+) or the equivalent amount of DMSO (Dox‐) for 48 hours, then the luciferase activity was monitored by a luminometer. Result was normalized to that of the control sample (*n* = 3). Error bars indicate the mean ± standard error (SE) *p < 0.05, **p < 0.01 by two‐tailed Student's *t*‐test

We first investigated whether RUNX family regulates *NFATC2* in T cells. From public chromatin immunoprecipitation sequencing (ChIP‐seq) data in activated mouse CD4 T cell, RUNX1 binds to promotor region of *IL2, IFNG, TNF* and *CSF2* within islands of active chromatin mark by H3K4 methylation and H3K27 acetylation (Figure S1A). Also, RUNX1 binds to *NFATC2* promotor region in CD4 T cell of mouse and human, and Jurkat T cell acute lymphoblastic leukemia cell line (Figure 1A). Since it has been shown that RUNX family members have redundant functions, next, we looked at the association between the whole *RUNX* family and *NFATC2* expression. Clustering of *RUNX1, RUNX2, RUNX3* and *NFATC2* gene expression data from published data of human CD4 T‐cells confirmed a cluster of *RUNX*‐family‐high and *NFATC2*‐high expression (Figure 1B). We found a weak but significant correlation between each *RUNX* family gene and *NFATC2* gene expression respectively (Figure 1C). Although not significant in RUNX2, reporter experiments showed *NFAT* promoter including RUNX consensus binding site (Figure S1B) increased its reporter activity by overexpression of each RUNX family (Figures 1D and S2). In addition, shRNA knockdown targeting the common region of *RUNX* family members (*PanRUNX*) of Jurkat cells resulted in reduced reporter activity of the *NFATC2* promoter (Figure 1E). These results suggest RUNX family have redundant roles in the expression of *NFATC2* in human T cells.

### 3.2 Targeting whole RUNX family reduces the expression of *NFATC2* and cytokine genes

**FIGURE 2:**
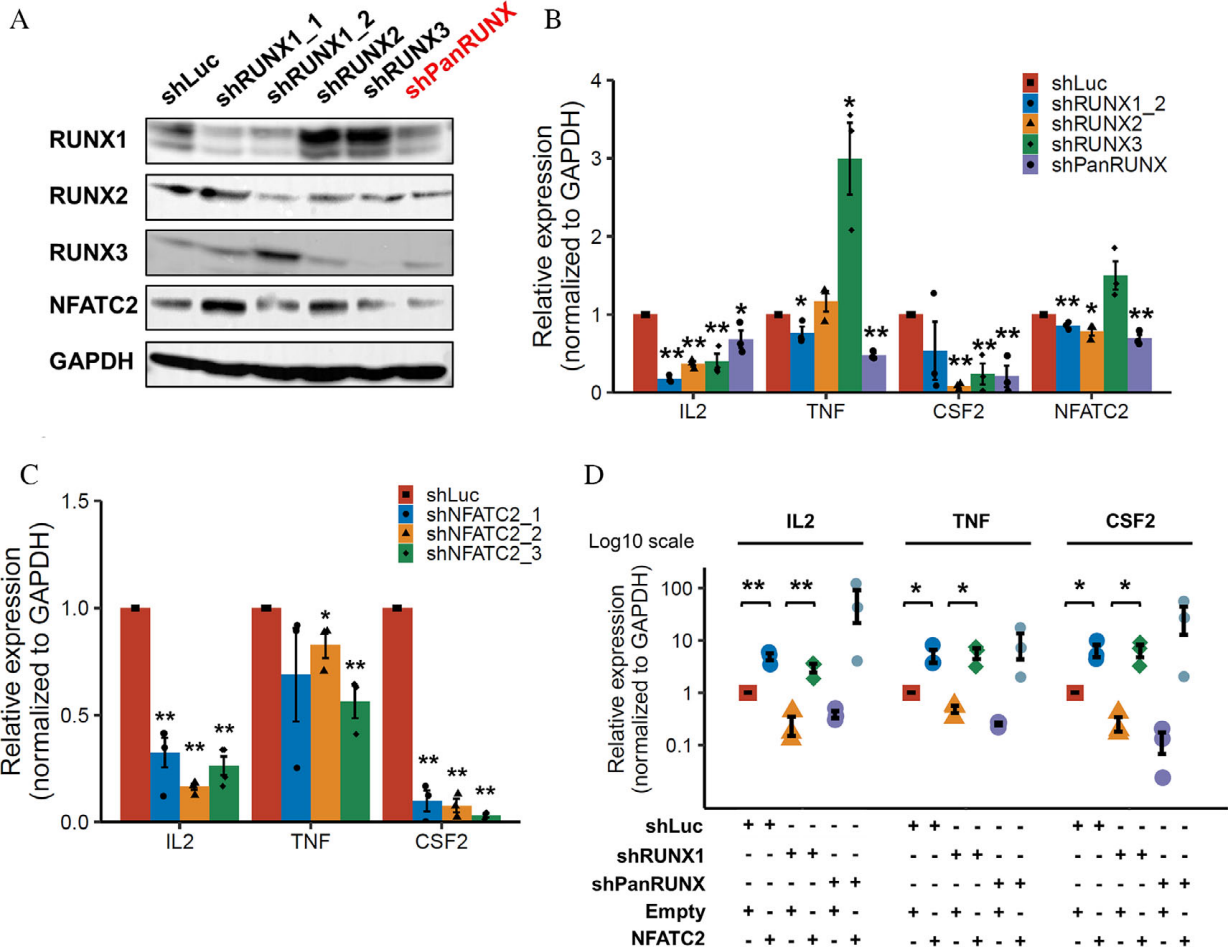
RUNX family knockdown reduces the expression of *NFATC2* and cytokine genes. (A) Down‐regulation of *NFATC2* expression upon RUNX inhibition. Jurkat cells were transduced with control (sh*Luc*) or with RUNX shRNAs (sh*RUNX1*_1, sh*RUNX1*_2, sh*RUNX2*, sh*RUNX3* and sh*PanRUNX*) and cultured in the presence of 3 μM doxycycline. Twenty‐four hours after treatment, cell lysates were processed for immunoblotting. (B and C) *RUNX* and *NFATC2*‐depletion‐mediated repression of cytokine gene expression. (B) *RUNX*‐depleted Jurkat cells were treated as in (A). (C) Jurkat cells were transduced with *NFATC2* shRNAs (sh*NFATC2*_1, sh*NFATC2*_2 and sh*NFATC2*_3) and cultured in the presence of 3 μM doxycycline. Twenty‐four hours after treatment, cells were activated by 50 ng/ml PMA and 1 μM ionomycin. Twelve hours after treatment, total RNA was prepared and analyzed by real‐time RT‐PCR. Values were normalized to that of control vector‐transduced cells (*n* =  3). (D) Restoring *NFATC2* expression in RUNX‐depleted Jurkat cells reverts RUNX‐depletion‐mediated inhibition of cytokine expression. Non‐RUNX‐depleted (sh*Luc*) and RUNX‐depleted (sh*RUNX1*_2 and sh*PanRUNX*) Jurkat cells transduced with (*NFATC2*) or without (Empty) lentivirus expressing *NFATC2*. Cells were treated with 3 μM doxycycline for 24 hours, then activated by 50 ng/ml PMA and 1 μM ionomycin. Twelve hours after treatment, total RNA was prepared and analyzed by real‐time RT‐PCR. Values were normalized to that of control vector‐transduced cells (*n* = 3). Error bars indicate the mean ± standard error (SE) *p < 0.05, **p < 0.01, compared with the control by two‐tailed Student's *t*‐test

To investigate whether RUNX is essential for the expression of *NFATC2* and cytokine genes, we silenced RUNX by shRNA‐knockdown of Jurkat cell line. *RUNX1* and *PanRUNX* knockdown in Jurkat cells suppressed the expression of *NFATC2* at the mRNA and protein level in Jurkat cells, most notably in *PanRUNX* knockdown. (Figures 2A and 2B). In addition, *PanRUNX* knockdown reduced *TNF* the most. In contrast, the knockdown of each single *RUNX* family members reduced *IL2* and *CSF2* expression, suggesting that the inhibition of these cytokines by *RUNX* inhibition can occur regardless of *NFATC2* inhibition. Knockdown of *NFATC2* suppressed gene expression of *IL2, IFNG* and *TNF* in Jurkat cells (Figures 2C and S3A). These were consistent with NFATC2 being a kye factor for cytokine expression in T cells. Restoring the expression of *NFATC2* in *RUNX*‐knocked down Jurkat cells reverted the *RUNX*‐depletion‐mediated cytokine inhibition (Figure 2D and S3B), suggesting that RUNX family members are enhancing but dispensable in initiating cytokine gene expression, which is consistent with previously reported results [33]. Taken together, these data collectively indicated that RUNX family member positively regulates Th1‐associated cytokine genes and *CSF2* expression through both enhancing cytokine genes and directly transactivating *NFATC2* expressions, supporting the potential of targeting all RUNX families as a strategy to reduce T cell activation.

### 3.3 RUNX inhibitor Chb‐M’ suppresses effector T cell response

**FIGURE 3:**
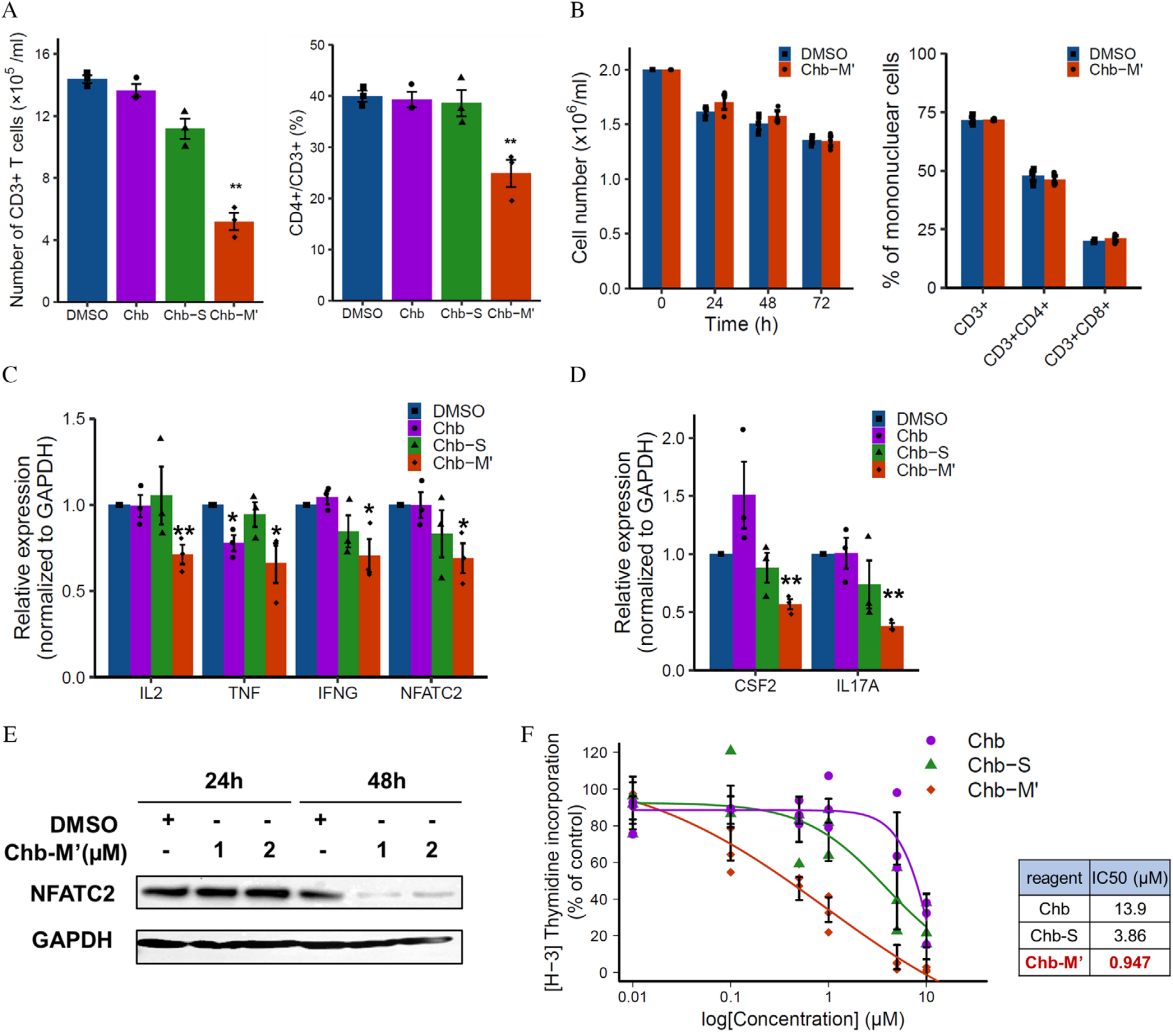
RUNX inhibitor, Chb‐M’, suppresses T‐cell proliferation and reduces the expression of *NFATC2* and cytokine genes. (A) Effect of Chb‐M’ on human T cell proliferation with OKT3‐mediated TCR activation. Human PBMCs (1.0×10^6^ /ml) were cultured with OKT3‐coated plate and 100 U/ml rIL2, and treated with 1μM Chb,1 μM Chb‐S, 1μM Chb‐M’ or the equivalent amount of DMSO. Four days after treatment, the viable CD3‐positive T cell number was counted by trypan blue staining, and the percentage of CD4‐positive T cells were analyzed by flow cytometry (*n* = 3). (B) Effect of Chb‐M’ on human T cell itself in cell viability and CD4 to CD8 ratio. Human PBMCs (2.0×10^6^ /ml) were treated with 1μM Chb‐M’ or the equivalent amount of DMSO. The viable total cell number was counted by trypan blue staining at 24, 48 and 72 hours after treatment, and the percentage of CD3, CD4 and CD8‐positive T cells were analyzed by flow cytometry at 48 hours after treatment (*n* = 3). (C and D) Effect of Chb‐M’ on cytokine genes and *NFATC2* mRNA expression. Human PBMCs were treated with 1μM Chb,1 μM Chb‐S, 1μM Chb‐M’ or the equivalent amount of DMSO. (C) Six hours after treatment, cells were activated by 50 ng/ml PMA and 1 μM ionomycin, and total RNA was prepared four hours after activation. (D) Simultaneously with treatment, cells were stimulated with OKT3‐coated plates, and total RNA was prepared twenty‐four hours after activation. Total RNA was analyzed by real‐time RT‐PCR for *IL2, TNF*, *IFNG* and *NFATC2* in (C) and *CSF2* and *IL17A* gene expression in (D). Values were normalized to that of control vector‐transduced cells (*n* =  3). (E) Effect of Chb‐M’ on NFATC2 expression. Jurkat cells were treated with 1 or 2 μM Chb‐M′ for 24 and 48 h, and then cell lysates were prepared and immunoblotted. Error bars indicate the mean ± standard error (SE) *p < 0.05, **p < 0.01 compared with the control (DMSO) by two‐tailed Student's *t*‐test. (F) Dose‐response curves of Chb, Chb‐S and Chb‐M’ in mixed lymphocyte reaction of human peripheral blood mononuclear cells. Cells were treated with the indicated concentrations of PI polyamides or Chb. Six days after treatment, cell proliferation was assayed by [^3^H] thymidine uptake assay. (*n* = 3). The table shows IC50 values of Chb, Chb‐S and Chb‐M’

We have previously reported a potent RUNX inhibitor Chb‐M’ (Figure S4A) and its efficacy in suppressing RUNX‐mediated gene expression [27]. In this study, we examined whether its RUNX inhibiting effect is applicable for suppression of effector T cell response through targeting the *RUNX‐NFATC2* axis. First, we first investigated whether Chb‐M' inhibits T‐cell receptor (TCR)‐dependent T cell proliferation. Chb‐M' inhibited OKT3‐stimulated TCR‐dependent proliferation and further reduced the percentage of CD4 in human PBMCs (Figure 3A). Through this we examined that, while Chb‐M’ showed significant T cell inhibiting effects on Jurkat cells, chlorambucil (Chb) itself or Chb‐conjugated PI polyamides that target scrambled DNA sequence (5′‐WGGCCW‐3′; Chb‐S, Figure S4B) were ineffective against these cells. In addition, Chb‐M’ had no effect on the viability of the T cells themselves or the CD4 to CD8 ratio (Figure 3B). In addition, Chb‐M’ significantly reduced cytokine genes and *NFATC2* mRNA expression in human T cells activated by PMA and ionomycin or OKT3‐induced stimulation (Figures 3C, 3D and S5), and reduced *NFATC2* expression at protein level in Jurkat cells (Figure 3E) compared to the DMSO control.

Next, for the study of alloreactive T‐cell response, we performed mixed lymphocyte reaction by mixing human PBMCs from different donors and [^3^H] thymidine uptake assay. Compared to control, Chb‐M’ significantly reduced alloreactive T cell proliferation (Figure 3F). These data collectively indicate that RUNX inhibition by Chb‐M’ could be a novel therapeutic strategy for suppressing effector T cell response.

### 3.4 Chb‐M’ ameliorates xenogeneic GVHD

**FIGURE 4:**
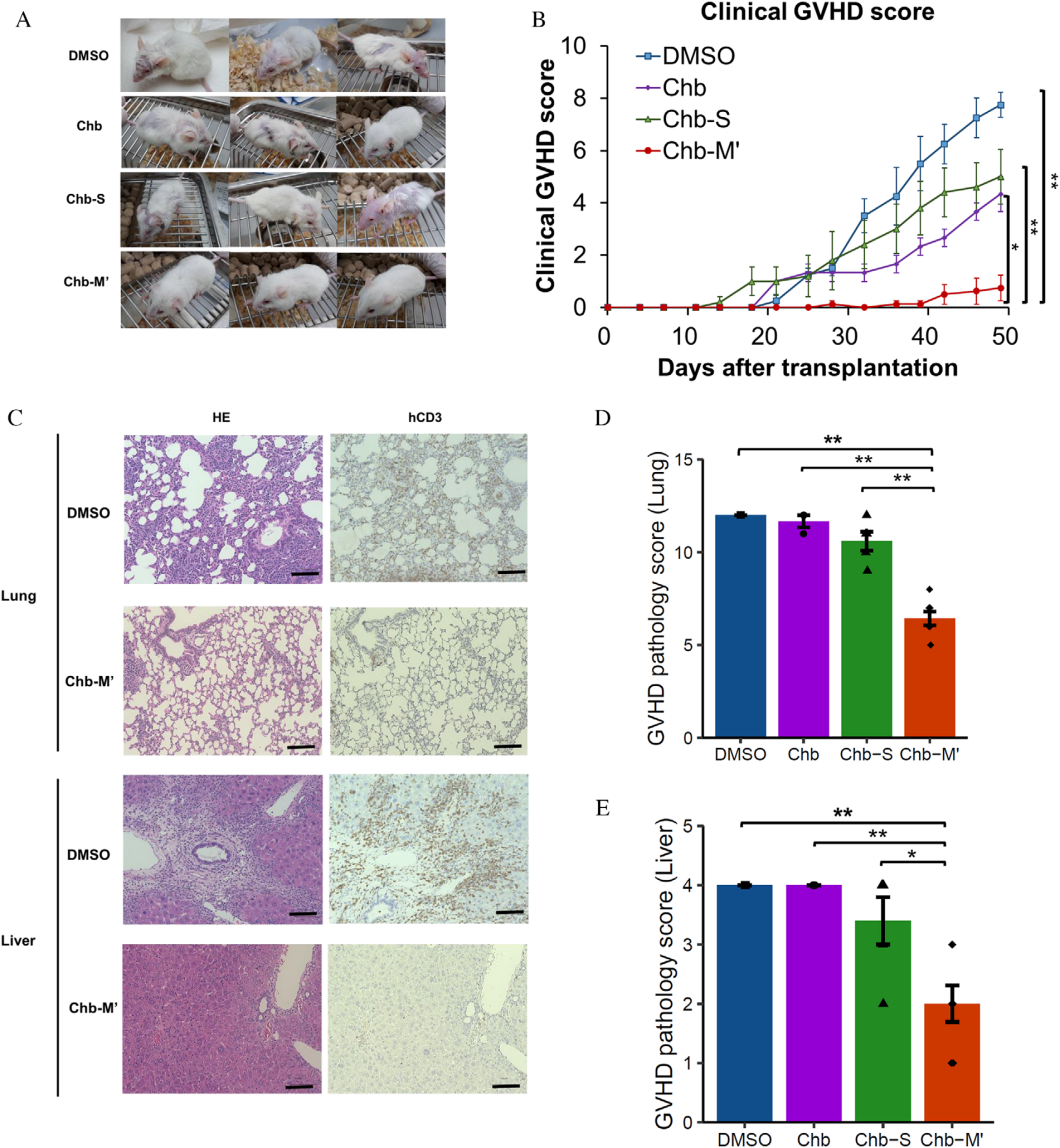
Chb‐M′ suppresses xenogeneic graft‐versus‐host disease. (A) The pictures of NOG mice transplanted with human peripheral blood mononuclear cells (PBMCs) 7 weeks after cell transplantation. Mice were treated with Chb‐M’ (320 μg/kg body weight, twice per week intravenous injections) or with the equivalent amount of either Chb, Chb‐S or DMSO twice a week from day0. (B) Clinical GVHD score changes of NOG mice after transplantation of human PBMCs treated with DMSO (*n* = 4), Chb (*n* = 3), Chb‐S (*n* = 5) and Chb‐M’ (*n* = 8). (C) Immunohistochemistry in organs from xenogeneic GVHD‐induced mice. Livers and lungs were obtained 7 weeks after transplantation. The sections were stained with hematoxylin and eosin (HE), and anti‐human CD3 antibody. 3,3‐diamino‐benzidine (DAB) substrate was used for color development. Scale bars: 100 μm. (D and E) Pathological GVHD score of (D) luns and (E) livers in xenogeneic‐GVHD induced NOG mice treated with DMSO (*n* = 3), Chb (*n* = 3), Chb‐S (*n* = 5) and Chb‐M’ (*n* = 7). Error bars indicate the mean ± standard error (SE). *p < 0.05, **p < 0.01, one‐way analysis of variance, followed by the Tukey post hoc test

**FIGURE 5:**
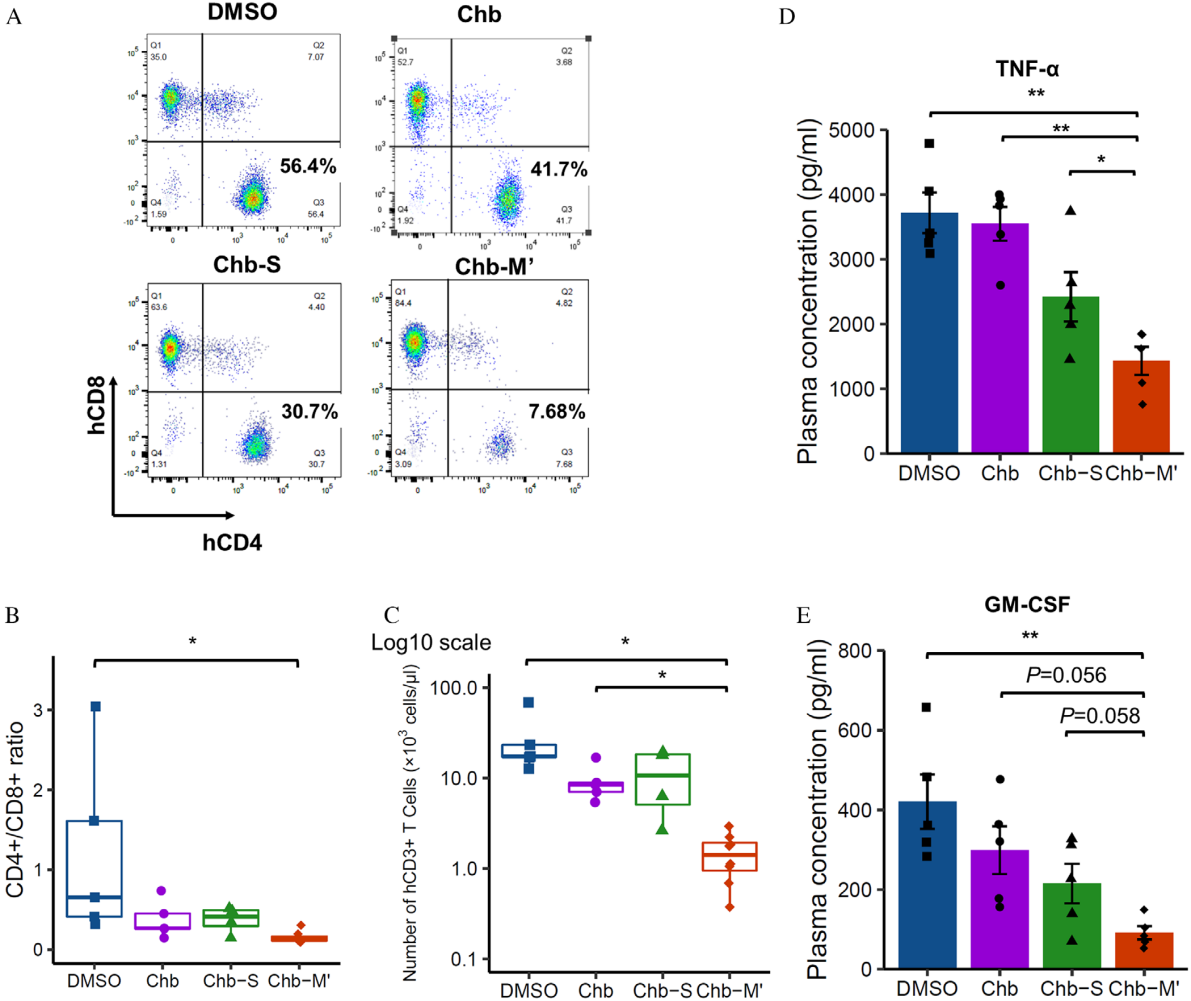
Chb‐M′ suppresses T cell proliferation and cytokine expression in vivo. Blood samples were collected from xenogeneic‐GVHD induced NOG mice treated with DMSO, Chb, Chb‐S and Chb‐M’ at week 4 after transplantation. (A) Flow cytometric data of PBMCs from each mouse. Peripheral blood mononuclear cells were stained with anti‐human CD45, CD3, CD4 and CD8 antibodies. (B) The ratio of CD4 to CD8 human T cells and (C) the number of human CD3 T cells of peripheral blood samples in each group of mice. Box plots in (B and C) show, median, quartiles (boxes), and range (whiskers). *p < 0.05, Kruskal–Wallis test, followed by Steel–Dwass multiple comparison. (D and E) Human (D) TNF‐α and (E) GM‐CSF cytokine concentrations of peripheral blood plasma from xenogeneic‐GVHD mice at week 4 after transplantation (*n* = 5 mice per group). Error bars indicate the mean ± standard error (SE). *p < 0.05, **p < 0.01, one‐way analysis of variance, followed by the Tukey post hoc test

Finally, we examined in vivo effect of Chb‐M’ for xenogeneic GVHD mouse model by transplanting human PBMC onto immunodeficient NOG mice. Compared to control, mice injected by Chb‐M’ showed almost no sign of GVHD assessed by clinical score (Figures 4A, 4B and S6). Lung and liver pathology of Chb‐M’‐injected mice showed that reduced human T cell infiltration and less pathogenic GVHD findings such as inflammation, pneumonitis and bile duct injury, compared to control group (Figures 4C and 4D). By analysing peripheral blood of GVHD mice, we found decreased T cell proliferation, especially CD4 T cell in Chb‐M’ injected mice (Figures 5A‐5C). Levels of TNF‐α and GM‐CSF, cytokines critically involved in GVHD pathology, were significantly reduced in the plasma of mice treated with Chb‐M’ compared with DMSO‐treated mice (Figures 5D, 5E and S7). These data collectively indicate that RUNX inhibition by Chb‐M’ could be a novel therapeutic strategy in GVHD.

## 4 DISCUSSION

Here, we demonstrate that inhibition of whole RUNX family suppresses *NFATC2* expression in human T cells, inhibits T cell proliferation and cytokine production, and the suppression of xenogeneic‐GVHD by RUNX inhibitor, Chb‐M’.

The important role that RUNX plays in T cell differentiation has been widely studied, but regulation of *NFATC2* expression by RUNX family in human T cells has been poorly studied to date. Although we presented data for public Chip‐seq data of only RUNX1 (Figure 1A), RUNX2 and RUNX3 can also bind to the same site because all RUNX family members share the RUNX‐binding consensus sequences 5′‐TGTGGT‐3′ or 5′‐TGCGGT‐3′. In addition, not only *RUNX1*, but also *RUNX2* and *RUNX3* showed a correlation between *NFATC2* and their gene expression levels in human CD4 T cells (Figures 1B and 1C), and reporter experiments showed that RUNX1‐3 increases the transcriptional activity of *NFATC2*, respectively (Figure 1D), indicating that RUNX1‐3 have redundant roles in the gene expression activity of *NFATC2*. Consistent with this, in knockdown experiments in Jurkat cells, *PanRUNX* knockdown reduced *NFATC2* expression the most than knockdown of each RUNX family alone (Figures 2A and 2B). Regarding the role of RUNX in gene expressions in activated naïve T cells, binding of RUNX1 to the enhancer is dependent upon prior induction of NFAT and AP‐1 [34, 35]. Indeed, knockdown of each RUNX family alone, even without suppression of *NFATC2* expression, could decrease in *IL2* and *CSF2* cytokines expression (Figures 2B). Otherwise, suppression of *TNF* expression was most evident with *PanRUNX* knockdown possibly through suppression of NFATC2 [36]. Considering the transactivation activity of RUNX family members in both cytokine genes and *NFATC2*, targeting whole RUNX family could be a strategy to suppress T cell activation rather more strongly than targeting either each single RUNX protein or NFAT alone.

Although the Th1 cell‐associated cytokines IFN‐γ, IL‐2 and TNF‐α have been implicated in the pathophysiology of acute GVHD, some studies have reported the opposite effects. This included their functional role in regulatory T cells (Treg) [37, 38, 39, 40], and therapeutic optimization in targeting these cytokines is a challenge that requires further efforts. Among GVHD‐associated cytokines, GM‐CSF blockade may be an attractive target candidate to prevent aGVHD [15, 16, 17]. The evidence for the regulation of *CSF2* gene expression by RUNX [23] and NFAT [41] has been established. Although the effects on the recruitment and activation of donor‐derived monocytes and dendritic cells, which are important in GVHD exacerbations [17], have not been analyzed, we have shown that Chb‐M' decreased the production of GM‐CSF expression in both in vitro experiments using primary human T cells and in an in vivo model of xenogeneic‐GVHD. Thus, while blocking therapy against GM‐CSF is being tested in clinical trials as a promising treatment for various inflammatory diseases [42, 43], our Chb‐M' may be one of the candidate drugs to target GM‐CSF.

In the vivo models that use our xenogeneic GVHD model in which human peripheral human T cells were injected into NOG mice, we showed that RUNX family inhibition by Chb‐M' inhibited T cell proliferation and GVHD phenomenon (Figures 4 and 5). Since RUNX1 and RUNX3 are play important roles in multiple stages of T‐cell development, targeting RUNX family may have various effects on T cell differentiation and effecter function in vivo. Previous reports have shown that in vivo analysis the *Runx1* or *Runx3* knockout mouse model is known to result in over‐immunity rather than suppression of the immune system. Deletion of *Runx1* in naive CD4+ T cells caused spontaneous cellular activation and cytokine production that eventually led to a catastrophic autoimmune inflammatory disease [44]. Besides, *Runx3* knockout mice spontaneously develop inflammatory bowel disease with enhanced expression of both Th1 and Th2 signature cytokines [45]. A possible reason for the discrepancy between these reports and our results may be that there is compensation by other RUNX families in a single *Runx* knockout model. Thus, we would like to emphasize that targeting all RUNX family can be effective in reducing suppressing effector T cell activation. We acknowledge that targeting whole RUNX family may also have a negative effect on T cell differentiation in humans, considering that mature thymocyte were virtually absent by reducing core‐binding factor β levels in mice, in which the activity of all three Runx proteins should be affected [46]. This is especially important in the context of HSCT, where there is a concern that T‐cell differentiation of transplanted donor cells in the thymus may be inhibited, so Chb‐M' may be better selected as an additional treatment for SR‐GVHD than for GVHD prophylaxis. And, in settings other than HSCT, it could be applied to diseases involving T cell activation such as autoimmune diseases.

We acknowledge that the study has several limitations. First, we only analyzed the effect of Chb‐M' on GVHD and not the graft‐versus‐leukemia (GVL) effect. The challenge in the current era is to prevent GVHD while sparing GVL effect. Donor CD8 T cells play a dominant role in mediating the GVL effect via cytotoxic T lymphocyte activity [47]. An essential role of Runx3 in cytotoxic function in activated mature CD8 T cells has been suggested by studies using *Runx3*‐deficient mice CD8+ T cells in vitro [48] and in vivo [49]. Thus, the therapeutic effect of GVHD with Chb‐M' may also be accompanied by suppression of GVL resulting in the risk of relapse of malignant disease. However, this may be offset by the antineoplastic effect of Chb‐M' depending on the type of malignant disease [27]. Secondly, despite the fact that RUNX1 is an interacting transcriptional partner for FOXP3 [21] and also regulates the expression of *FOXP3* in Tregs [50], we did not examine the effect of Chb‐M' on Tregs, which are critical mediators of immune tolerance after allogenic HSCT. Although it is possible that Chb‐M' may cause suppression of regulatory T cell production as well as conventional T cells, one strategy may be to combine low‐dose IL2 treatment with Chb‐M’, which has been used in recent years to restore Treg in clinical practice [37]. The effect of Chb‐M’ on GVL and Tregs will be the subject of further studies.

In conclusion, we have shown that targeting the whole RUNX family suppresses *NFATC2* expression and various cytokine gene expressions in T cells. Considering Chb‐M’ is expected to have therapeutic effects through a mechanism different from other existing GVHD drugs, the first priority is to test whether it is effective as an additional treatment for refractory GVHD in the clinical settings. Further studies are awaited to validate this new strategy against GVHD.

## CONFLICT OF INTEREST

The authors have no conflicting financial interests.

## AUTHORS CONTRIBUTIONS

Hirohito Kubota performed experiments, analyzed data and wrote the manuscript. Tatsuya Masuda, Mina Noura,Kana Furuichi and Masahiro Hirata performed experiments and analyzed data. Hidefumi Hiramatsu, Takahiro Yasumi, Tatsutoshi Nakahata, Yoichi Imai, Junko Takita and Souichi Adachi participated in discussions and the interpretation of data and results and commented on the research direction. Hiroshi Sugiyama synthesized and designed the PI polyamides. Yasuhiko Kamikubo designed and initiated the study, supervised research, and gave the final approval for submission.

## Supporting information

Supporting Information
