# Supplementary material for: RUNX inhibitor suppresses graft‐versus‐host disease through targeting RUNX‐NFATC2 axis
Source: EJHaem. 2021 May 19;2(3):449–58. doi: 10.1002/jha2.230 (PMC9175814; doi:10.1002/jha2.230)
Supplement: Supplementary file 1 — Supporting Information [file JHA2-2-449-s001.docx]

**Supplementary Information**

**Title: RUNX inhibitor suppresses graft-versus-host disease through targeting *RUNX-NFATC2* axis**

Authors: Hirohito Kubota^*^, Tatsuya Masuda, Mina Noura, Kana Furuichi, Hidemasa Matsuo, Masahiro Hirata, Tatsuki R. Kataoka, Hidefumi Hiramatsu, Takahiro Yasumi, Tatsutoshi Nakahata, Yoichi Imai, Junko Takita, Souichi Adachi, Hiroshi Sugiyama and Yasuhiko Kamikubo

Supplemental Figures p. 2

Supplemental Tables p. 10

Supplemental Methods p. 13

References p. 19

**Supplemental Figures**

**Supplemental Figure S1**

**
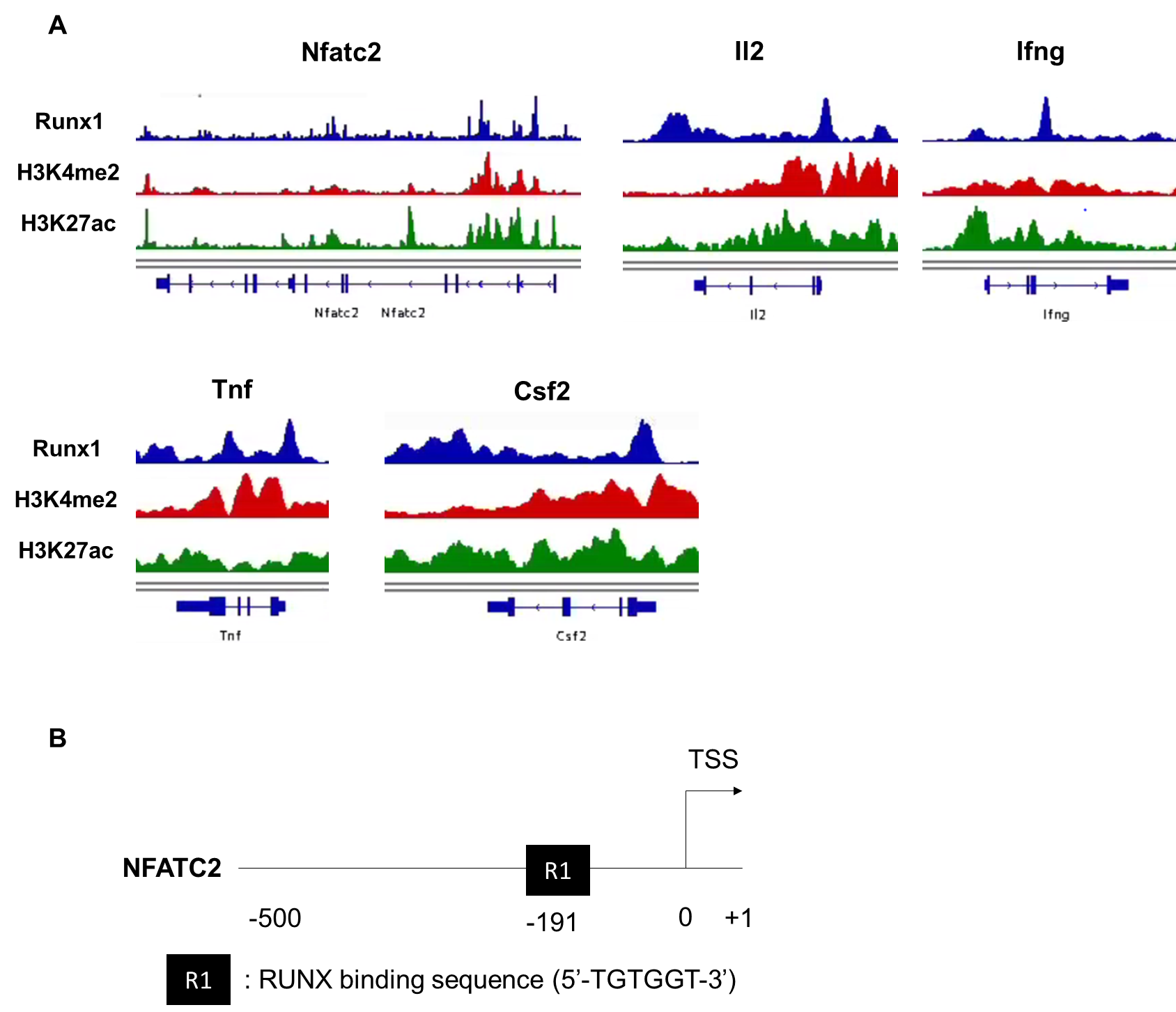
**

(A) RUNX-dependent transactivation of cytokine gene expression in mouse activated T cells. IGV snapshots showing the alignment data (BigWig or Wig format) around the *Nfatc2, Il2, Ifng, Tnf* and *Csf2* gene loci for Runx1, H3K4me2, H3K4me3, and H3K27ac ChIP-seq experiments in primary mouse CD4 T cell activated by PMA. (B) Proximal regulatory region (-500 bp to +1 bp) relative to transcription start site (TSS) of *NFATC2*.

**Supplemental Figure S2**

**
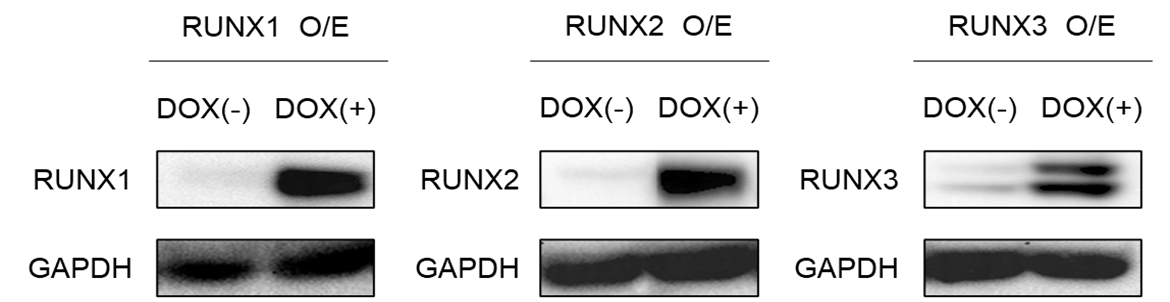
**

RUNX family overexpression in HEK293T cells. Immunoblotting of RUNX1, RUNX2, RUNX3 and GAPDH in HEK293 cells transduced with *RUNX1, RUNX2* and *RUNX3* expression lentiviral vectors (RUNX1 O/E, RUNX2 O/E and RUNX3 O/E). Cells were treated with 3 µM doxycycline [Dox (+)] or the equivalent amount of DMSO [Dox (-)] for 48 hours.

**Supplemental Figure S3**


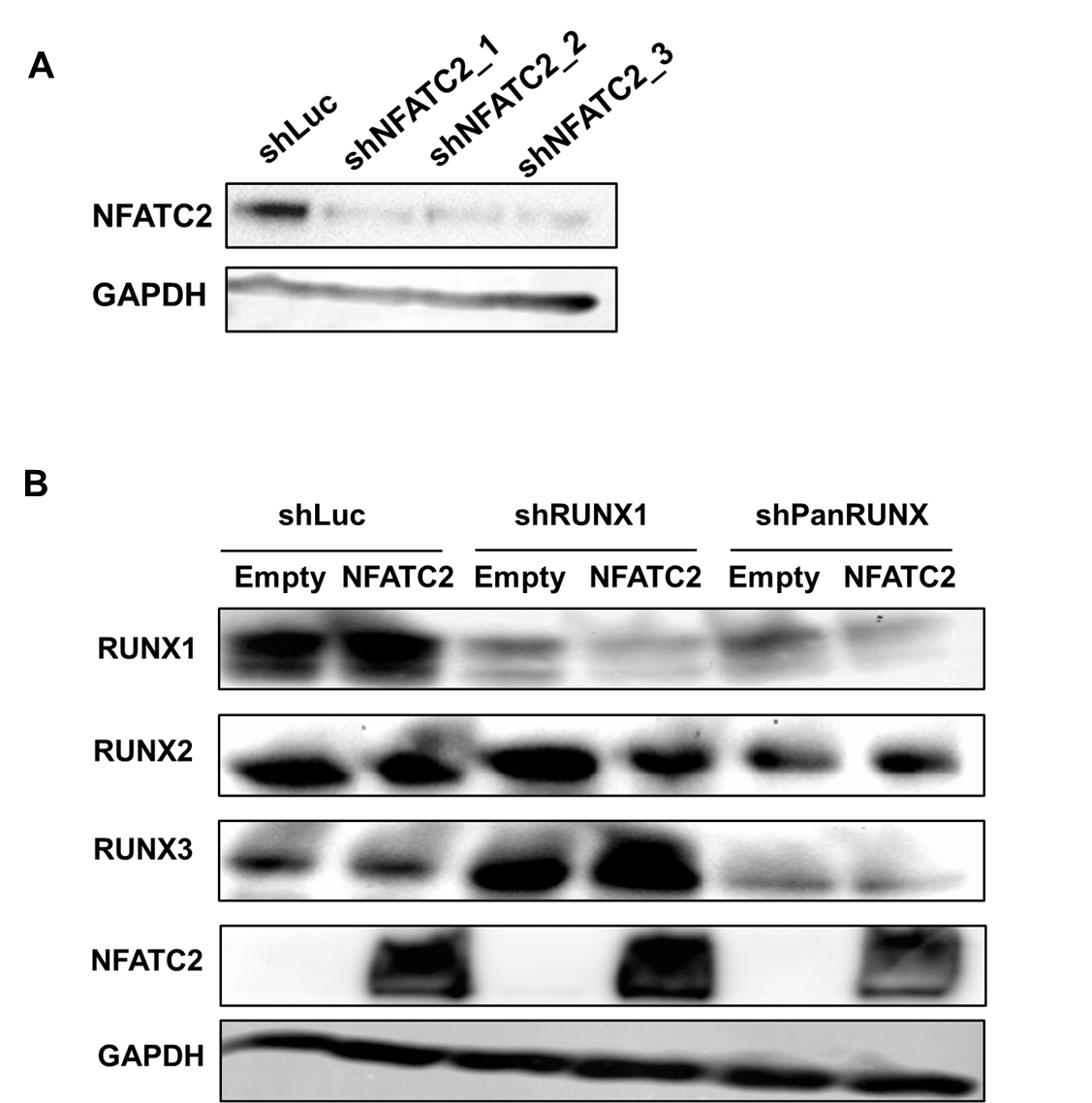


(A) NFATC2 knockdown in Jurkat cells. Immunoblotting of NFATC2 and GAPDH in Jurkat cells transduced with control (shLuc) or *NFATC2* shRNAs (sh*NFATC2*_1, sh*NFATC2*_2 and sh*NFATC2*_3). Cells were treated with 3 µM doxycycline for 24 hours. (B) NFATC2 rescue in Jurkat cells with shRNA knockdown of *RUNX1* and *PanRUNX*. Immunoblotting of RUNX1, RUNX2, RUNX3 and GAPDH in Jurkat cells transduced with control (sh*Luc*) , *RUNX1* shRNAs (sh*RUNX1*) and *PanRUNX* shRNAs (sh*PanRUNX*) with or without NFATC2 overexpression. Cells were treated with 3µM doxycycline for 48 hours.

**Supplemental Figure S4**

**
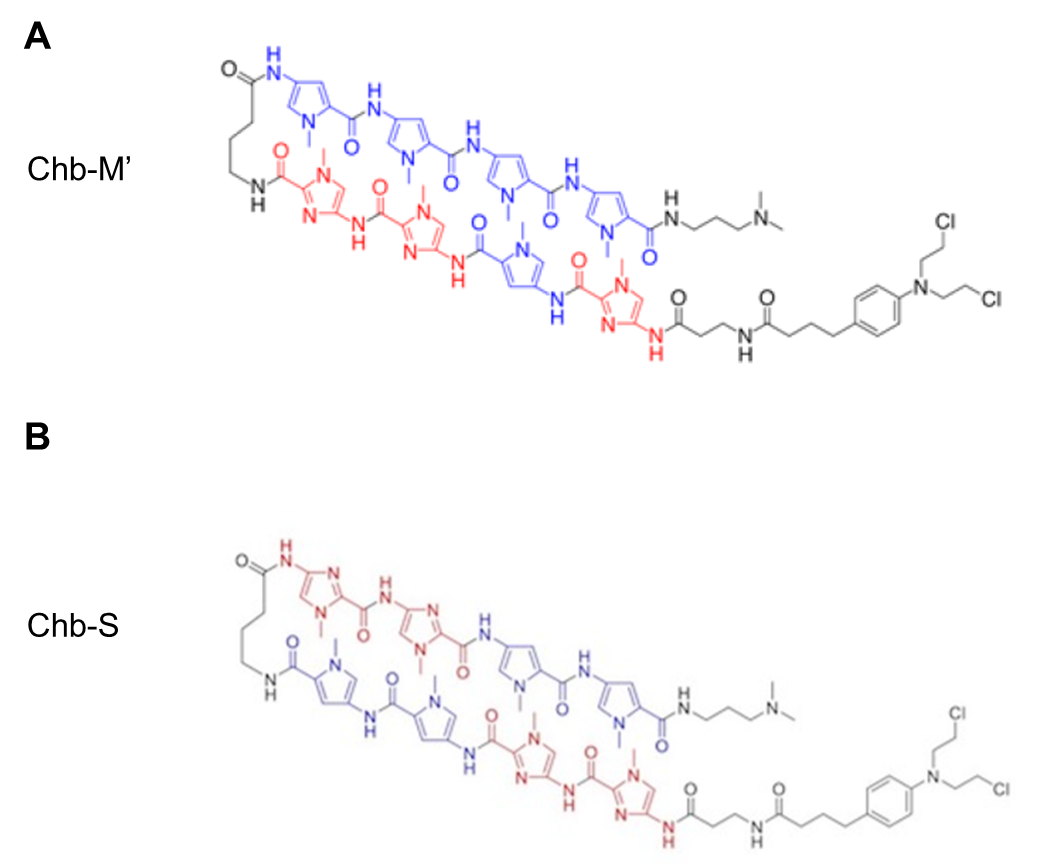
**

Chemical structure of the synthetic chlorambucil - PI polyamide conjugates (A) targeting 5’-TGTGGT-3’ (Chb-M’) and (B) targeting 5’-TGGCCT-3’ (Chb-S).

**Supplemental Figure S5**

**
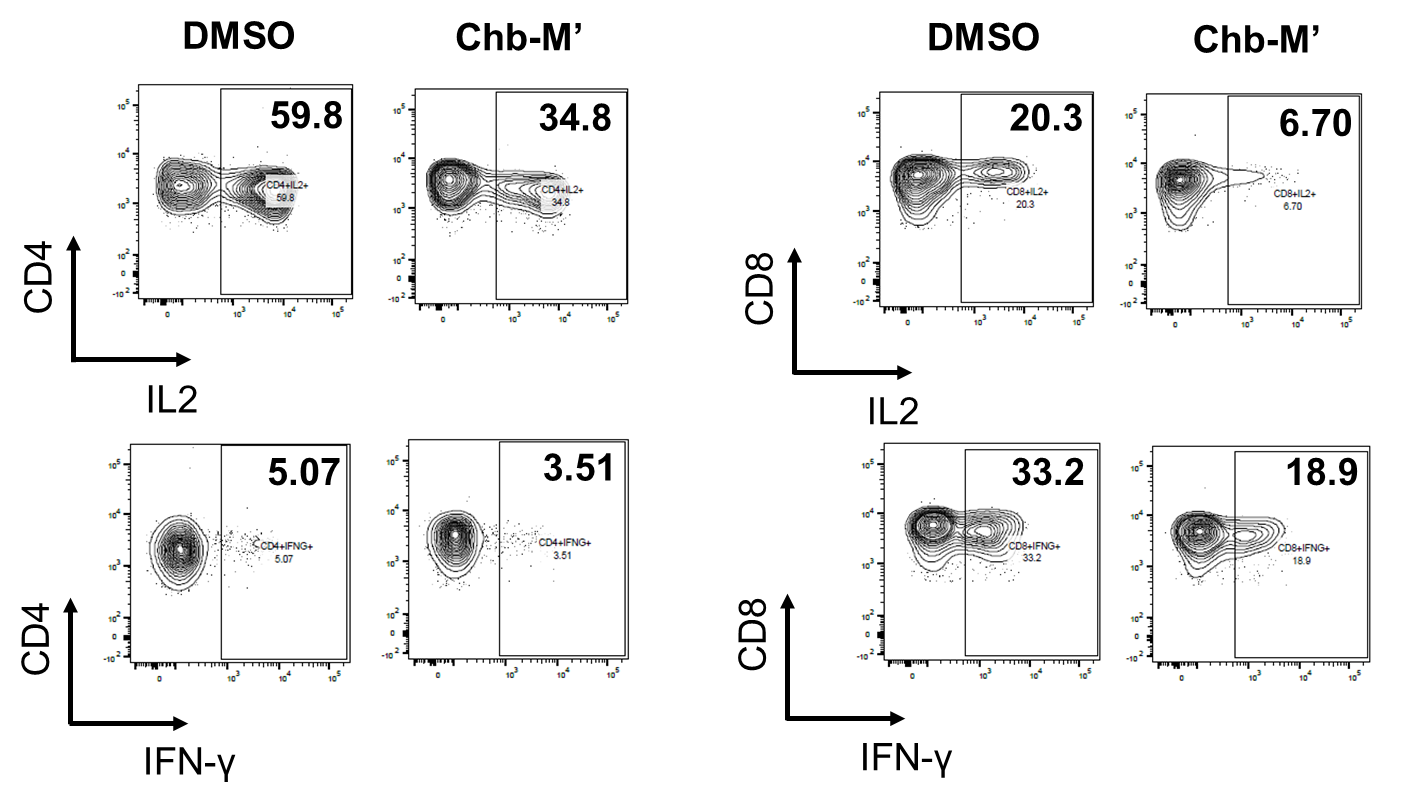
**

Contour plots show the IL2 and IFN-γ positive CD4 and CD8 positive activated T cells treated with DMSO or Chb-M’. Human peripheral blood mononuclear cells were treated with 1µM Chb-M’ or the equivalent amount of DMSO. Six hours after treatment, cells were stimulated by 50 ng/ml PMA and 1 µM ionomycin. Four hours after stimulation, cells were processed for intracellurar IL2 and IFN-γ staining and analyzed by flow cytometry.

**Supplemental Figure S6**

**
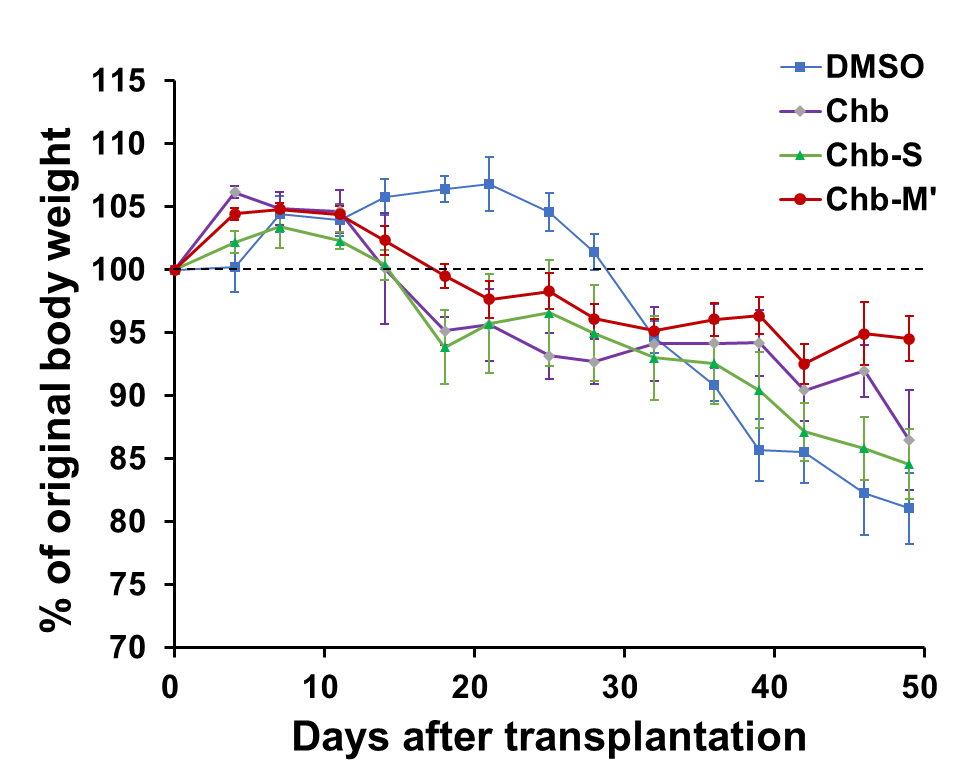
**

Body weight changes of NOG mice after transplantation of human peripheral blood mononuclear cells treated with DMSO (n=4), Chb (n=3), Chb-S (n=5) and Chb-M’ (n=8). Error bars indicate the mean ± standard error (SE). **P < 0.01 ,one-way analysis of variance, followed by the Tukey post hoc test.

**Supplemental Figure S7**


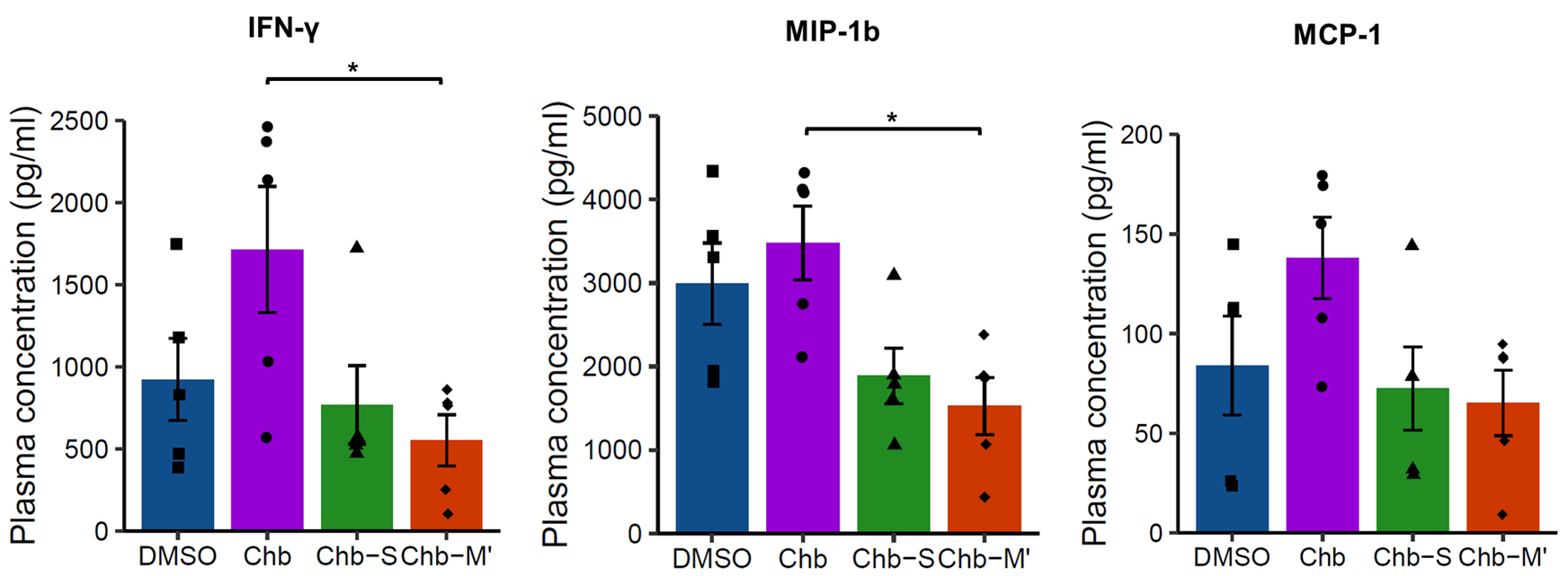


Human IFN-γ、MIP-1b and MCP-1 concentrations of peripheral blood serum from xenogeneic-GVHD mice at week4 after transplantation (n = 5 mice per group). Error bars indicate the mean ± standard error (SE). *P < 0.05, one-way analysis of variance, followed by the Tukey post hoc test.

**Supplemental Tables**

**Supplemental Table S1**

**Target sequences for shRNA knockdown experiments.**

|  | **Targets 5’ → 3’** |
| --- | --- |
| sh*Luc*. | CGTACGCGGAATACTTCGA |
| sh*RUNX1_*1 | AGCTTCACTCTGACCATCA |
| sh*RUNX1_*2 | AACCTCGAAGACATCGGCA |
| sh*RUNX2* | AAGGTTCAACGATCTGAGATTT |
| sh*RUNX3* | AAGCAGCTATGAATCCATTGT |
| sh*PanRUNX* | ACCGACAGCCCCAACTTCC |
| sh*NFATC2_*1 | GCAGAATCGTCTCTTTACA |
| sh*NFATC2_2* | GAATGACTGTGATCTTTGA |
| sh*NFATC2_3* | GTCAGTCAGGCTCTTACGA |

**Supplemental Table S2**

**PCR primers used for RT -qPCR**

|  | **Forward (5’ → 3’)** | **Reverse (5’ → 3’)** |
| --- | --- | --- |
| *IL2* | CCAAGAAGGCCACAGAACTGAA | TGCTGTCTCATCAGCATATTCACA |
| *IFNG* | GTGGAGACCATCAAGGAAGACA | TGGACATTCAAGTCAGTTACCGAA |
| *TNF* | AGGCAGTCAGATCATCTTCTCG | TATCTCTCAGCTCCACGCCA |
| *CSF2* | CTCAGAAATGTTTGACCTCCAG | TGACAAGCAGAAAGTCCTTCAG |
| *IL17A* | ATCTCCACCGCAATGAGGAC | CACCAGTATCTTCTCCAGCCG |
| *NFATC2* | CACGCGGTAGAGAAGACGG | CAGTCTGAACGCAGCCTCTC |
| *GAPDH* | CATGTTCGTCATGGGGTGAACCA | AGTGATGGCATGGACTGTGGTCAT |

**Supplemental Methods**

**ChIP-Seq data analysis**

RUNX1, H3K4me3 and H3K427ac Chip-seq datasets in human naïve CD4 T cells (SRA accession numbers SRX212444, SRX1142269, SRX1286408, respectively), RUNX1, H3K4me2, H3K4me3 and H3K427ac in Jurkat cells (SRA accession numbers: SRX2014504, SRX2022904, SRX7785397, SRX5974500, respectively) were downloaded from ChIP-Atlas^1^ (https://chip-atlas.org/). RUNX1, H3K4me3 and H3K427ac Chip-seq datasets in mouse activated CD4 T cells^2^ (GEO accession numbers: GSM1646846, GSM1646837, GSM1646839, respectively) were downloaded from the Gene Expression Omnibus (GEO) database of the National Center for Biotechnology Information (NCBI). Bigwig or wig format of Chip-seq datasets were visualized in the Integrative Genomics Viewer (IGV) genome browser.^3^

**CD4 T cell gene expression analysis**

Public gene expression data of human CD4 T cell samples (n=376) was downloaded from Immuno-Navigator^4^ (https://genomics.virus.kyoto-u.ac.jp/immuno-navigator/). Heat map analysis of the Z score of gene expression was carried out using the pheatmap R package with euclidean distance and Ward linkage method as distance measure and clustering methods. The Spearman correlation of gene expression was calculated by the R cor.test() function, and visualized with the ggplot R package.

**siRNA interference.**

Specific shRNAs targeting human *RUNX1, RUNX2, RUNX3, PanRUNX* and *NFATC2* were designed and subcloned into pENTR4-H1tetOx1 and CS-RfA-ETV (RIKEN BioResource Center, Ibaraki, Japan). Non-targeting control shRNA was designed against luciferase (shLuc). The target sequences were provided in Supplementary Table S1.

**Expression plasmids**

We amplified cDNAs for human *RUNX1, RUNX2, RUNX3,* and human *NFATC2* cDNA (FLJ09127AAAN) were obtained from NBRC (NITE Biological Resource Center, Kisarazu, Japan). They were inserted into CSIV-TRE-Ubc-KT expression vectors, which were a gift from Hiroyuki Miyoshi (RIKEN BioResource Center). All of the PCR products were verified by DNA sequencing. For the tetracycline-inducible gene or shRNA expression, doxycycline was added to the cell culture at a final concentration of 3 μM.

**Production and transduction of lentivirus**

To produce lentivirus, HEK293T cells were transiently cotransfected with lentivirus vectors such as psPAX2 and pMD2.G by polyethylenimine (PEI, Sigma-Aldrich). Forty-eight hours after transfection, viral supernatants were collected and immediately used for infection, and then successfully transduced cells were sorted using a FACSAria III (BD Biosciences).

**Luciferase reporter assay.**

Putative region of *NFATC2* (−500 bp to 0 bp of transcription start site) was cloned from the human genomic DNA using the following primers: F 5′- CTAGGCGCTGGGGAGTTGAG -3′ and R 5′- ACGGCGGCGCGAGCTTCCTG -3′, and then subcloned into pGL4.20 [luc2/Puro] vector (Promega, Madison, WI, USA). Both pGL4.20 NFATC2 promoter vector and pRL-CMV control vector (TOYOBO, Osaka, Japan) were co-transfected into HEK293T cells that are stably expressing expression vector of *RUNX1, RUNX2, and RUNX3*, as well as Jurkat cells that are stably expressing shRNA targeting *PanRUNX*. Promoter activities were measured using Dual-Glo Luciferase Assay System (Promega) and detected by Spark multimode microplate reader (Tecan, Zurich, Switzerland) according to the manufacturer’s instructions.

**Immunoblotting**

Immunoblotting was conducted as previously described.^5^ Briefly, cells were washed twice in ice-cold phosphate-buffered saline and lysed in lysis buffer. Whole cell extracts were separated by SDS-polyacrylamide gel electrophoresis, and electrotransferred onto polyvinylidene difluoride membranes. Membranes were probed with the following primary antibodies: anti-RUNX1 (A-2, Santa Cruz Biotechnology, Dallas, TX, USA), anti-RUNX2 (Cell Signaling Technology, Beverly, MA, USA. clone D1H7), anti-RUNX3 (Cell Signaling Technology, clone D6E2), anti-NFATC2 (A-2, Santa Cruz Biotechnology), and anti-GAPDH (FL-335, Santa Cruz Biotechnology). HRP-conjugated anti-rabbit IgG and anti-mouse IgG (#7074 and #7076, Cell Signaling Technology) were used as secondary antibodies. Blots were visualized using Chemi-Lumi One Super (Nacalai Tesque, Inc. Kyoto, Japan) and the ChemiDoc XRS+ Imager (Bio-Rad Laboratories, Hercules, CA, USA) according to the manufacturers’ recommendations.

**RT-qPCR**

Total RNA was isolated with RNeasy Mini Kit (QIAGEN, Valencia, CA, USA) and reverse transcribed with ReverTra Ace kit (TOYOBO) to generate cDNA. RT-qPCR mixtures were prepared using the Power SYBR Green Master Mix (Applied Biosystems, Waltham, MA, USA) and qPCR was carried out with the Step one plus Real-Time PCR System (Applied Biosystems) according to the manufacturer’s instructions. The results were normalized to *GAPDH* levels. Relative expression levels were calculated using the 2-ΔΔCt method. Primers used for RT-qPCR are listed in Supplemental Table S2.

**Flow cytometry**

The monoclonal antibodies used in the flow cytometric analyses were fluorescein isothiocyanate (FITC)-conjugated anti-human CD3 (eBioscience, San Diego, CA, USA), allophycocyanin (APC)-conjugated anti-human CD4 (BD Biosciences), phycoerythrin (PE)-conjugated anti-human CD8 (BD Biosciences), Brilliant Violet 421–conjugated anti-CD8 (BioLegend, San Diego, CA, USA), FITC-conjugated anti-human CD45 (BD Biosciences), APC-conjugated anti-mouse CD45 (BD Biosciences), PE–cy7-conjugated anti-human IL2 (eBioscience), and PE–conjugated anti-human IFN-γ (eBioscience). For intracellular cytokine staining, cells were stimulated in the presence of Brefeldin A (GolgiPlug, BD Biosciences, San Jose, CA, USA), and were ﬁxed in Cytoﬁx/Cytoperm (BD Biosciences) and washed in Perm-wash (BD Biosciences). After blocking, cells were stained with the antibodies and then washed in Permwash. Samples were analyzed using a BD FACS Verse and BD FACSuite software according to the manufacturer’s protocol. The data were analyzed using FlowJo version 10 software (Tree Star, Ashland, OR, USA).

**Mixed lymphocyte reaction**

Peripheral blood mononuclear cells were isolated from two healthy people and resuspended in RPMI1640 with 10% FCS and 1% P/S. Stimulating cells were treated with 25 ng/ml mitomycin C for 30 minutes at 37°C to abolish their capacity to proliferate and to make the reaction unidirectional. Mixed lymphocyte cultures were carried out in round-bottomed 96-well plates with 1×10^5^ responder cells and 1×10^5^ stimulating cells in a volume of 0.1 ml with the drugs in the concentrations indicated and incubated for 6 days. [^3^H] thymidine (PerkinElmer, Inc. Waltham, MA, USA) was then added (1 uCi/well) in 50 µl culture medium and incubated for 18 hours. The samples were harvested in the UniFilter FilterMate Harvester (PerkinElmer, Inc). [^3^H] -thymidine incorporation of the samples was measured using MicroBeta 2 microplate scintillation counter (PerkinElmer, Inc). Curves of percent inhibition of dimethyl sulfoxide (DMSO) -treated control were drawn, and IC50 of the indicated compounds were calculated by the drc R package.

**Immunohistochemistry.**

Morphological (using hematoxylin and eosin [H&E] staining) and immunohistochemical analysis were applied in the murine tissue sections of lung and liver. The organs were harvested, fixed with 4% paraformaldehyde, and embedded in paraffin. Immunohistochemistry (IHC) was performed using antibodies directed against human CD3 antigen by rabbit anti-human CD3 (Leica Biosystems, San Diego, CA, USA) . The tissue section images were captured using BZ-9000 All-in-One Fluorescence Microscope (Keyence, Osaka, Japan).

**Measurement of cytokine in plasma**

Plasma samples were analyzed for cytokines using the Bio-Plex Pro Human Cytokine 17-plex Assay (Bio-Rad Laboratories, Hercules, CA, USA) according to the manufacturer’s instructions. Plasma samples of each mice were diluted 2-fold with standard dilution solution and stored. Cytokine/chemokine concentrations were measured using BioPlex MAGPIX Multiplex Reader (Bio-Rad Laboratories) and calculated using a standard curve.

**Synthesis of PI polyamides.**

Synthesis of Chb-M’ was conducted as previously reported.^6^ Briefly, Py-Im polyamide supported by oxime resin was prepared in a stepwise reaction by Fmoc solid-phase protocol. The product with oxime resin was cleaved with *N*,*N*-dimethyl-1,3-propane diamine (1.0 mL) at 45 °C for 3 hours. The residue was dissolved in the minimum amount of dichloromethane and washed with dimethyl ether to yield a 59.6 mg. To the crude compound (59.6 mg, 48.1 μmol), a solution of chlorambucil (32.6 mg, 107 μmol), benzotriazole-1-yl-oxy-tris-pyrrolidino-phosphonium hexafluorophosphate (PyBOP) (101 mg, 195 μmol) and *N*,*N*-diisopropylethylamine (100 μL, 581 μmol) in *N*,*N*-dimethylformamide (DMF) (300 μL) was added. The reaction mixture was incubated for 1.5 hours at room temperature, washed with dimethyl ether and DMF for three times and dried in vacuo. The crude product was purified by reversed-phase flash column chromatography (water with 0.1% trifluoroacetic acid/MeCN). After lyophilization, product was obtained (30.2 mg, 19.8 μmol). Machine-assisted polyamide syntheses were performed on a PSSM-8 (Shimadzu) system with computer-assisted operation. Flash column purifications were performed by a CombiFlash Rf (Teledyne Isco, Inc.) with C18 RediSep Rf Flash Column. Electrospray ionizationtime-of-flight mass spectrometer by using positive ionization mode and proton nuclear magnetic resonance (1H NMR) spectra were recorded with a JEOL JNM ECA-600 spectrometer operating at 600 MHz and in parts per million (ppm) downfield relative to tetramethylsilane used as an internal standard to verify the quality of synthesized PI polyamides.

**Statistics**

Statistical analysis was conducted using R version 3.5.3 (http://www.R-project.org/). Differences in mean values between two groups were analyzed by Student’s t-test. The results were represented as the average ± SEM values obtained from at least three independent experiments. In vivo experiments, one-way analysis of variance followed by the Tukey post hoc test was used for parametric data, and Kruskal–Wallis test followed by Steel–Dwass multiple comparison was used for non-parametric data to compare multiple groups. Values of P <0.05 were considered statistically significant.

**References**

1. Oki S, Ohta T, Shioi G, Hatanaka H, Ogasawara O, Okuda Y, et al. ChIP-Atlas: a data-mining suite powered by full integration of public ChIP-seq data. EMBO Rep. 2018 Dec;19(12).

2. Bevington SL, Cauchy P, Piper J, Bertrand E, Lalli N, Jarvis RC, et al. Inducible chromatin priming is associated with the establishment of immunological memory in T cells. EMBO J. 2016;35(5):515–35.

3. Robinson JT, Thorvaldsdóttir H, Winckler W, Guttman M, Lander ES, Getz G, et al. Integrative genomics viewer. Vol. 29, Nature biotechnology. 2011. p. 24–6.

4. Vandenbon A, Dinh VH, Mikami N, Kitagawa Y, Teraguchi S, Ohkura N, et al. Immuno-Navigator, a batch-corrected coexpression database, reveals cell type-specific gene networks in the immune system. Proc Natl Acad Sci [Internet]. 2016 Apr 26;113(17):E2393 LP-E2402. Available from: http://www.pnas.org/content/113/17/E2393.abstract

5. Morita K, Masamoto Y, Kataoka K, Koya J, Kagoya Y, Yashiroda H, et al. BAALC potentiates oncogenic ERK pathway through interactions with MEKK1 and KLF4. Leukemia [Internet]. 2015;29(11):2248–56. Available from: https://doi.org/10.1038/leu.2015.137

6. Morita K, Suzuki K, Maeda S, Matsuo A, Mitsuda Y, Tokushige C, et al. Genetic regulation of the RUNX transcription factor family has antitumor effects. J Clin Invest. 2017;127(7):2815–28.
